# Ecotoxicological Study of Tannic Acid on Soil and Water Non-Target Indicators and Its Impact on Fluvial and Edaphic Communities

**DOI:** 10.3390/plants12234041

**Published:** 2023-11-30

**Authors:** María Rosa Pino-Otín, Guillermo Lorca, Jonatan Val, Natalia Ferrando, Diego Ballestero, Elisa Langa

**Affiliations:** Universidad San Jorge, Autovía Mudéjar, km. 299, 50830 Villanueva de Gállego, Zaragoza, Spain; rpino@usj.es (M.R.P.-O.); glorca@usj.es (G.L.); jovalpu@gmail.com (J.V.); nferrando@usj.es (N.F.); dballestero@usj.es (D.B.)

**Keywords:** tannic acid, soil ecotoxicity, water ecotoxicity, fluvial communities, edaphic communities

## Abstract

Tannic acid (TA) is a key tannin extensively used in the leather industry, contributing to around 90% of global leather production. This practice leads to the generation of highly polluting effluents, causing environmental harm to aquatic ecosystems. Additionally, tannins like TA degrade slowly under natural conditions. Despite efforts to reduce pollutant effluents, limited attention has been devoted to the direct environmental impact of tannins. Moreover, TA has garnered increased attention mainly due to its applications as an antibacterial agent and anti-carcinogenic compound. However, our understanding of its ecotoxicological effects remains incomplete. This study addresses this knowledge gap by assessing the ecotoxicity of TA on non-target indicator organisms in both water (*Vibrio fischeri*, *Daphnia magna*) and soil environments (*Eisenia foetida*, *Allium cepa*), as well as natural fluvial and edaphic communities, including periphyton. Our findings offer valuable insights into TA’s ecotoxicological impact across various trophic levels, underscoring the need for more comprehensive investigations in complex ecosystems. Our results demonstrate that TA exhibits ecotoxicity towards specific non-target aquatic organisms, particularly *V. fischeri* and *D. magna*, and phytotoxicity on *A. cepa*. The severity of these effects varies, with *V. fischeri* being the most sensitive, followed by *D. magna* and *A. cepa*. However, the soil-dwelling invertebrate *E. foetida* shows resistance to the tested TA concentrations. Furthermore, our research reveals that substantial TA concentrations are required to reduce the growth of river microbial communities. Metabolic changes, particularly in amino acid and amine metabolism, are observed at lower concentrations. Notably, the photosynthetic yield of river periphyton remains unaffected, even at higher concentrations. In contrast, soil microbial communities exhibit greater sensitivity, with significant alterations in population growth and metabolic profiles at a very low concentration of 0.2 mg/L for all metabolites. In summary, this study offers valuable insights into the ecotoxicological effects of TA on both aquatic and terrestrial environments. It underscores the importance of considering a variety of non-target organisms and complex communities when assessing the environmental implications of this compound.

## 1. Introduction

Tannins, among which tannic acid (TA) is found, are mostly used in the leather industry. Around 90% of global leather undergoes a process where tannins are used in combination with chromium salts [1,2,3], and according to Kanth et al. [4], between 350,000 and 400,000 tons of vegetable tannins are spent on leather processing every year [4]. This industry is especially relevant in developing countries, like Brazil [5] or Morocco [6]. Effluents from this process are highly pollutant because of these compounds [7]. There are many studies reporting the environmental damage provoked by these effluents on fish, invertebrates, bacteria, or algae [6,8,9,10,11,12,13,14].

Apart from effluents, this industry discards 40–50% of tanned leather, producing about 600,000 tons of solid waste per year worldwide [15], which constitutes one more source of surface and groundwater [16,17,18,19] and soil pollution [15]. In fact, in a simulation study performed by Qiao et al. [15], where the migration of leather tannins and chromium salts in soils was studied, they found two interesting facts: (i) tannins slowly degraded in natural conditions and (ii) the total chromium leaching efficiency was promoted by tannins in the leachates. This means that this kind of lixiviate and the previously cited effluents deserve more attention from an ecotoxicological point of view [15,20]. 

To generate less pollutant effluents, many researchers focus their investigations on the replacement of chrome–tannin complexes with other tannin–chrome-free structures [21,22]. However, none of these studies have inquired into the effect of tannins, themselves, in the environment. In addition to the presence of tannins in leather-processing by-products, they are also found in waste from the cork [23] and food industries [24].

When studying tannins from different points of view, TA is their representative compound [24,25,26,27,28,29]. TA is a water-soluble gallotannin that belongs to the hydrolysable class of tannins [30]. It is a secondary metabolite of many plants, which can be found in natural surface waters [31,32]. 

TA has received increasing interest in recent years. Searching in the Web of Science for the term ’tannic acid’ in the ‘topic’ field, the number of results rose from 4628 in the first decade of the 2000s (2000–2009) to 14,844 in the second decade (2010–2020), with most of them concentrated in the following areas: agriculture, plant sciences, chemistry and biochemistry, pharmacology, food science and technology, environmental sciences, and nutrition.

This growing interest is focused, for instance, on its antibacterial activity alone or incorporated in nanoparticles, biofilms, or other biostructures [33,34,35,36,37,38,39], its anti-carcinogenic properties [40,41,42], its heavy metal removal capacity in wastewater [43], and its role in antibiotic soil sorption [29] or degradation in aquatic environments [27]. 

Whatever the application, TA will end up in the environment in one way or another. The fact that it is a natural compound does not mean there is an absence of ecotoxicity, as previously demonstrated with other natural products [44]. There are some studies that shed some light on the toxicity of TA in some aquatic organisms. Zhao et al. [45], for example, found significant toxicity in *Cytophaga columnaris* fish species. Goel et al. [46] studied the hepatotoxic effects of TA in two teleost fish, Varanka et al. [47] assessed the toxic effects in *Cyprinus carpio* fish, and Xie et al. [48] examined the same effects in zebrafish and Saha et al. [49] in *Oreochromis mossambicus* fish, as well as chronic effects after 90 days’ exposure. Although limited, some literature is available about the toxicity of TA in soil biomarkers, for example, on legumes [50]. 

However, there is still no complete ecotoxicological study of it, a need that Pautou already pointed out in 2000 [28]. So, these findings suggest that TA may also affect ecosystems at higher levels, highlighting the need for further study in complex communities.

Therefore, the objective of this study was to evaluate the ecotoxicity of TA in the environment using non-target indicator organisms in water and soil, as well as in fluvial and edaphic natural communities, in order to have a realistic and complete view through the different levels of the trophic chains of these environments.

## 2. Results

### 2.1. Genetic Analysis of Microbial Populations

#### 2.1.1. River Communities

**Figure 1:**
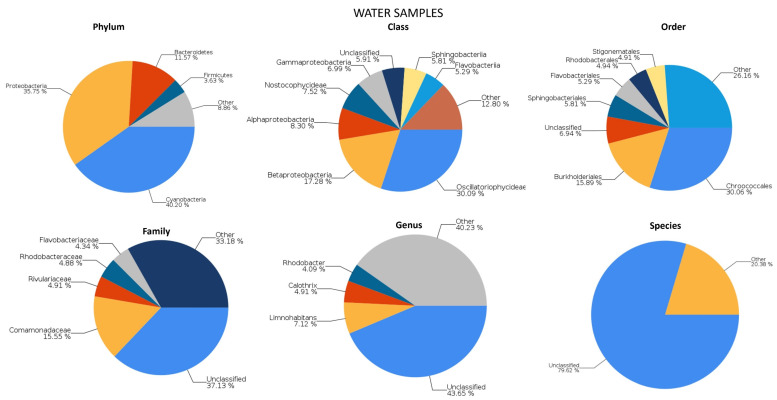
Phylum, class, order, family, genus, and species classification results for water samples.

Phylogenetic analysis of the DNA sequences of the 16 S rRNA genes led to the identification of the taxonomic classification of the microorganisms in our river samples, which can be seen in the graphs in Figure 1.

The percentage of total reads classified in the different taxonomic levels, including kingdom, phylum, class, and order, were in the range of 99.59% to 93.06%. However, the sequencing was less successful for family, genus, and species, with percentages of 62.87, 56.35, and 20.38%, respectively. In Figure 1, the top taxa abundance of different taxonomic levels can be seen.

The dominant phyla were Cyanobacteria, Proteobacteria, and, to a lesser extent, Bacteroidetes, which is a highly representative pattern of freshwater environments [51]. 

Among Cyanobacteria, the predominant class was Oscillatoriophycideae (over one-third of cyanobacteria), which belong to the order Chroococcales, a dominant cyanobacterial group in freshwater ecosystems [52], characterized by coccoid cells often surrounded by a mucilaginous envelope [53].

The Beta- and Alphaproteobacteria classes are usually the most abundant Proteobacterias in bacterial communities in freshwater followed by Gammaproteobacteria [54], as we found in our samples. Within the Betaproteobacteria, almost all of them belonged to the order Burkholderiales, which is prevalent in various rivers [55,56], and to the family Commamonadaceae, the most abundant family found in our samples. The order Rhodobacterales (practically all of them being Rhodobacteriaceae) dominated among Alphaproteobacteria. This order was found to be predominant in other rivers in Spain [57].

The classes Flavobacteria and Sphingobacteria were the most abundant within the Bacteroidetes phylum, similarly to other studies in river waters [58,59,60]. 

#### 2.1.2. Soil Communities

Kingdom, phylum, class, order, and family were optimally sequenced (above 81% of taxa), and only genus and species were identified below this ratio (76.85 and 36.10%, respectively).

**Figure 2:**
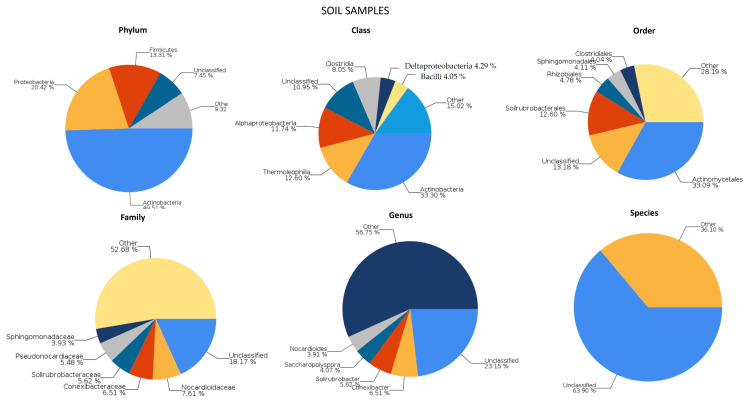
Phylum, class, order, family, genus, and species classification results for soil samples.

As can be seen in Figure 2, the predominant phylum identified was Actinobacteria (approximately half of bacterial reads), followed by Proteobacteria and Firmicutes, which is a type and proportion of microorganisms characteristic of many edaphic ecosystems [51,61,62].

Actinobacteria are an important phylum of common Gram-positive bacteria in soil and plant matter [63]. More than half of the Actinobacterias identified in our samples belong to the order Actinomycetales, with two families identified: Nocardioidaceae and Pseudonocardiaceae. Actinomycetales are a ubiquitous order in soil environments [64,65,66]. The class Thermoleophilia (all of them belonging to the order Solirubrobacteales) had the second highest abundance in the phylum Actinobacteria and also have been identified in different types of soils [67].

The main class of Proteobacteria identified was Alphaproteobacteria, followed by Deltaproteobacteria and Gammaproteobacteria. The predominant orders of these families are Rhizobiales, Myxococcales, and Xanthomonadales, respectively. However, the three classes show a great diversity at the level of family, genus, and species, resulting in fewer clearly dominant groups.

The phylum Firmicutes, also frequent in different types of soils [68,69,70,71,72,73] and in soils in Spain similar to that studied here [74], has the class Clostridia (with two main orders, Clostridiales) as the most abundant in our samples.

### 2.2. Average Well Color Development (AWCD) of Microbial Populations

#### 2.2.1. Water Samples

**Figure 3:**
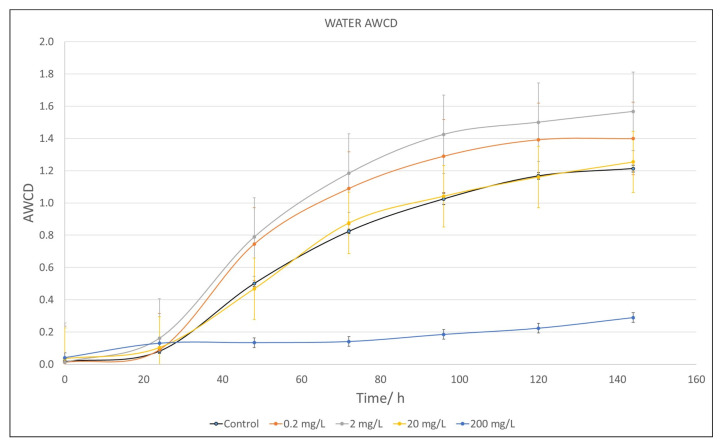
Average well color development (AWCD) of metabolized substrate in Biolog EcoPlates^®^ by river water microorganisms after 144 h exposure to different concentrations of tannic acid (TA) (shown at the bottom of the figure). Values are compared to a control value as reference (river communities not treated with TA). Each point is an average of three replicates and includes the error bars representing their standard deviation.

Figure 3 shows the impact of increasing concentrations of TA on the growth of bacterial communities from a river water sample for a 144 h incubation period in Biolog Ecoplates^®^ (Hayward, CA, USA). Only the highest dose (200 mg/L) provoked a significative decrease in AWCD (*p* < 0.05) with respect to the control (black line).

#### 2.2.2. Soil Samples

**Figure 4:**
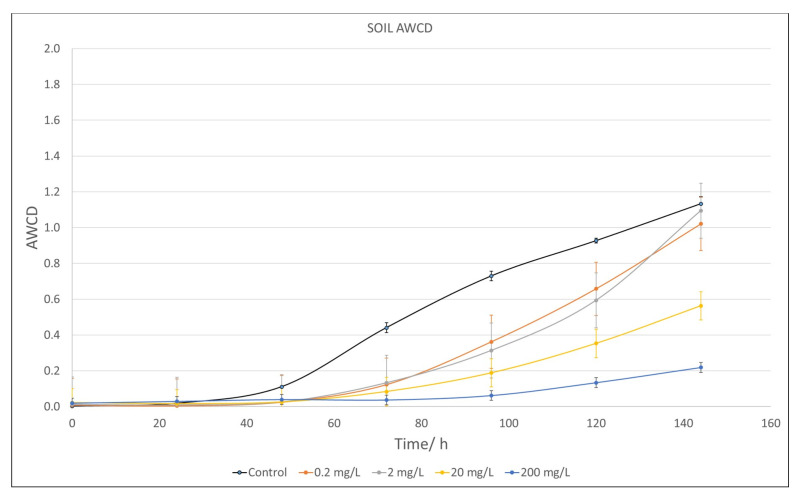
Average well color development (AWCD) of metabolized substrate in Biolog EcoPlates^®^ by soil microorganisms after 144 h exposure to different concentrations of TA (shown at the bottom of the figure). Values are compared to a control value as reference (soil communities not treated with TA). Each point is an average of three replicates and includes the error bars representing their standard deviation.

The behavior of the AWCD with time (144 h) for the microbial communities obtained from soil samples exposed to different concentrations of TA is illustrated in Figure 4. As can be seen, the effect of TA on the soil communities was stronger than on the river water ones. In this case, every concentration showed lower microbial growth than the control curve (black). This decrease was easily detectable as early as 0.2 mg/L, even though only 200 mg/L of TA showed significant differences with respect to the control (*p* < 0.05).

### 2.3. Community-Level Physiological Profiling (CLPP)

#### 2.3.1. Water Samples

**Figure 5:**
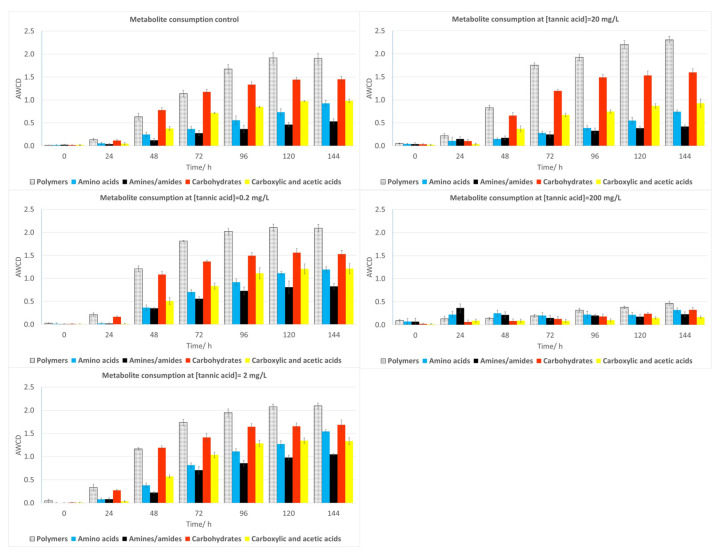
Metabolic profile of water samples after 144 h exposure to TA. Bars represent the AWCD growth for each group of metabolites of water bacteria. Each value is an average of three replicates and includes the error bars representing their standard deviation.

Figure 5 gives information about changes in the metabolic profile of river water microorganisms at different TA concentrations when they are grouped according to the type of the carbon source with which they were supplemented in BIOLOGs ecoplates^®^, that is, polymers, carbohydrates, carboxylic and acetic acids, amino acids, and amines, for 144 h. The first bar graph represents the control (river microorganisms not exposed to TA).

For the lowest concentrations (0.2 and 2 mg/L), no significant changes were observed for any group, although at 20 mg/L, the microbial capacity to metabolize amines and amino acids showed a small decrease, which, however, was only significant (*p* < 0.05) for polymers and carboxylic and acetic acids at the highest concentration (200 mg/L).

#### 2.3.2. Soil Samples

**Figure 6:**
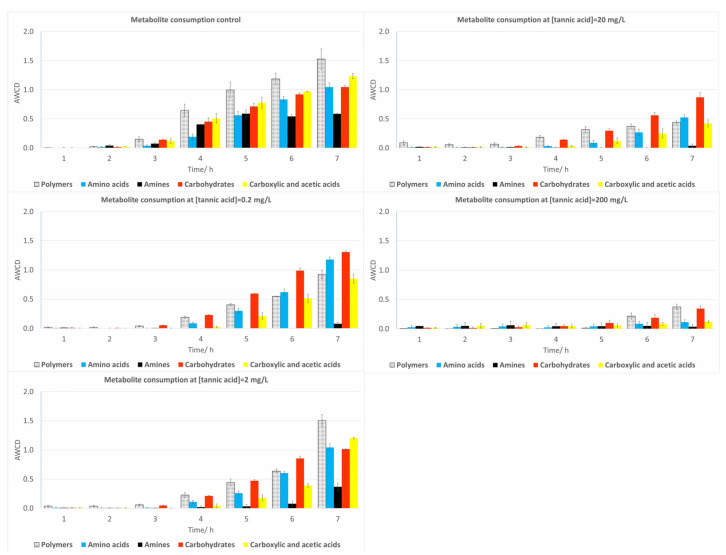
Metabolic profile of soil samples after 144 h exposure to TA. Bars represent the AWCD growth for each group of metabolites of soil bacteria. Each value is an average of three replicates and includes the error bars representing their standard deviation.

Information about changes in the physiological profiling of the soil microorganisms with time at the TA doses tested is provided in Figure 6 for the five classes of metabolites used in Biolog Ecoplates^®^. As can be seen, soil microorganisms were more sensitive to TA exposure than river microorganisms. 

In general terms, the ability of the soil microorganisms to metabolize substrates was reduced with higher TA concentrations for the cited metabolite classes, reaching almost no metabolic consumption for the highest concentration (200 mg/L). The most significant changes (*p* < 0.05) were detected for polymers, carboxylic and acetic acids, and amines at 200 mg/L.

### 2.4. Periphyton

No effect of TA was observed on periphyton species at the concentrations tested (Supplementary Information Figure S1), with LC_50_ > 1000 mg/L and LC_10_ = 7.02 (CI:0.36–46.44) mg/L.

### 2.5. Daphnia magna

*Daphnia magna* serves as a robust indicator of water quality due to its exposure to toxins via two pathways: surface contact and ingestion as a filter feeder, making it highly sensitive to environmental changes. Additionally, its swift reproductive cycle, widespread presence in diverse aquatic environments, and significant role in the food chain—being a staple for numerous aquatic organisms—underscore its importance. Its reaction to contaminants not only informs us about the quality of the water but also about the potential impact of the contaminants on the broader ecosystem [75].

**Figure 7:**
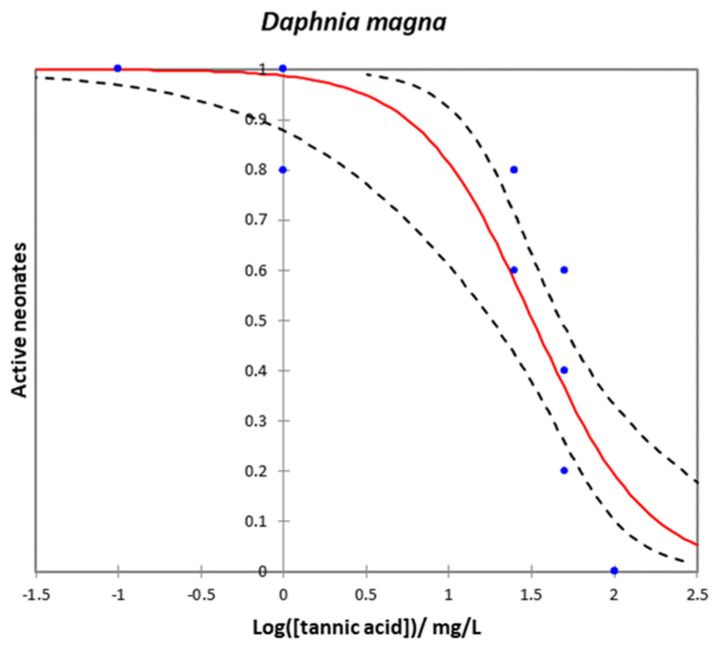
Dose–response curve for Daphnia magna test after 24 h–exposure to TA. Curves are the average of 5 replicates. Red line is the model, blue dots are experimental data, and dashed lines are the inferior and superior confidence limits (95%).

Figure 7 shows the dose–response curve for the D. magna immobilization test after 24 h exposure to TA. The results were analyzed with a chi-squared test, indicating high significance (*p* < 0.0001). TA influenced D. magna’s mobility, with EC_50_ and EC_10_ values of 32.038 mg/L (CI: 18.101–47.441 mg/L) and 5.606 mg/L (CI: 0.699–11.784 mg/L), respectively.

### 2.6. Vibrio fischeri

The *Vibrio fischeri* assay is a straightforward and economical method commonly used for screening and evaluating a diverse array of potentially harmful substances in ecotoxicology. It is known for its high sensitivity, ease of use, reliability, and consistency, making it a convenient addition to a test battery employed in assessing the ecotoxicity of various compounds. However, this assay, as is the case for D. magna, does not accurately reflect the chronic or long-term effects of contaminants [76].

**Figure 8:**
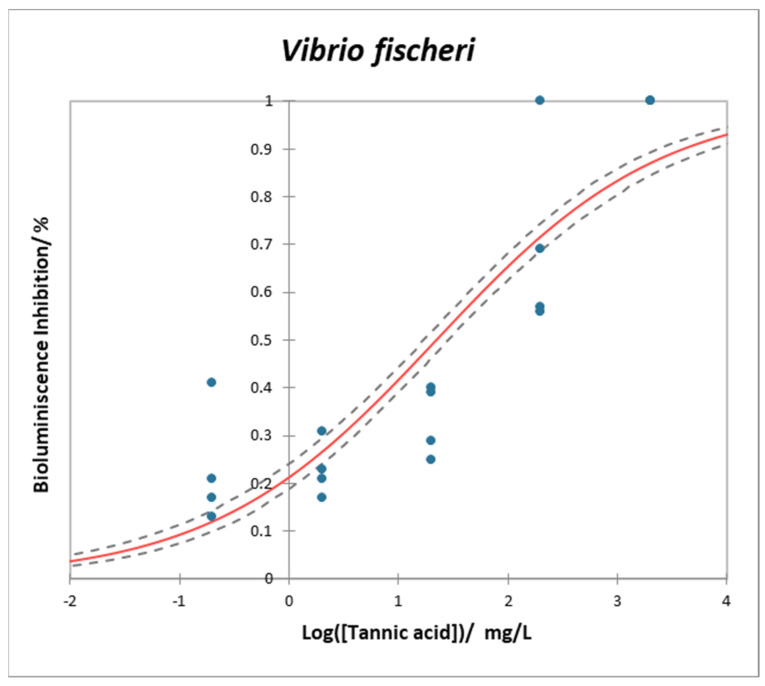
Dose–response curve for the bioluminescence assay for *Vibrio fischeri* after 24 h–exposure to TA. Curves are the average of 4 replicates. Red line is the model, blue dots are experimental data, and dashed lines are the inferior and superior confidence limits (95%).

The dose–response curve for the bioluminescence assay carried out on *V. fischeri* is displayed in Figure 8. A significance of *p* < 0.0001 was obtained when the dose–response values were analyzed. The EC_50_ and EC_10_ values were 22.000 mg/L (CI: 17.075–28.444 mg/L) and 0.121 mg/L (CI 0.028–0.198 mg/L), respectively.

### 2.7. Eisenia foetida

*Eisenia foetida* activity significantly impacts soil health, nutrient cycling, and overall ecosystem productivity in terrestrial environments. Their roles as decomposers, soil engineers, and contributors to nutrient cycling make them crucial in maintaining healthy and productive ecosystems [77]. *E. foetida* is commonly employed in terrestrial ecotoxicology [78,79] due to its high sensitivity as an indicator of soil quality and the possibility of conducting long-term assays. This species is exposed to contaminants in the soil through two pathways: firstly, through the skin via a thin epidermal cuticle and a glandular orifice that connects the worms with the surrounding environment, and secondly, through ingestion while burrowing and stirring the soil.

*E. foetida* survival was not influenced by any concentration of TA within the range studied (Supplementary Information Figure S2) with LC_50_ > 2000 mg/L.

### 2.8. Allium cepa

Finally, the *Allium cepa* root growth assay has also been frequently used [80,81,82] as a phytotoxicity test due to its sensitivity, swift response, ease of handling, and representation of effects on higher plants.

**Figure 9:**
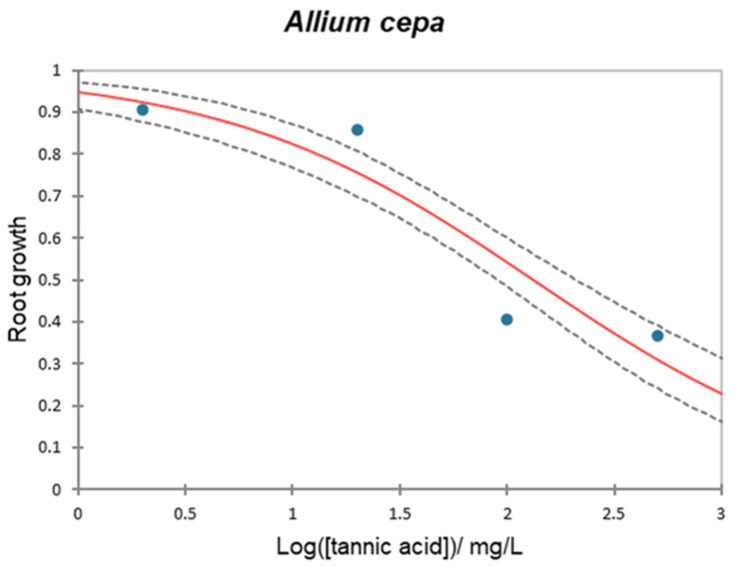
Dose–response curve for *Allium cepa* test after 72 h exposure to TA. Curves are the average of 12 replicates. Red line is the model, blue dots are experimental data, and dashed lines are the inferior and superior confidence limits (95%).

Figure 9 offers the dose–response curve for *A. cepa* after 72 h of incubation with TA. *A. cepa* root elongation was modified when exposed to the TA concentrations. The EC_50_ value was 133.389 mg/L (CI: 90.364–210.467 mg/L) and EC_10_ value was 3.415 mg/L (CI: 1.236–6.693 mg/L). The results’ significance was evaluated using a chi-squared test, which showed high significance (*p* < 0.0001).

## 3. Discussion

Our results show that TA exhibits ecotoxicity towards individual non-target water organisms such as *V. fischeri* and *D. magna*, as well as phytotoxicity towards *A. cepa*. From most to least ecotoxic (according to their LC_50_ values), the order is as follows: *V. fischeri* > *D. magna* > *A. cepa*. However, it did not display toxicity on the soil invertebrate *E. foetida* at the concentrations tested.

Our study also reveals that very high concentrations of TA are needed to produce a reduction in the growth of the river microbial population (of the order of 200 mg/L), although at 20 mg/L, some changes can be seen in the metabolic profile compared to the control, especially in the ability to metabolize amino acids and amines. There were also no changes in the photosynthetic yield of the river periphyton at the highest concentrations tested (100 mg/L). However, soil microbial communities appeared to be more sensitive to TA, modifying population growth with an intense change in the metabolic profile at 0.2 mg/L for all metabolites.

### 3.1. The Effects of TA on Water Bioindicators and Water Ecosystems

*D. magna* is a planktonic crustacean that is used as a highly reliable indicator for testing water quality contamination. It is a filter-feeding organism that feeds by straining suspended particles from the water and, thus, accumulating potential contaminants. Although it is unlikely that TA can cross membranes via simple diffusion due to its physicochemical properties, such as its high molecular weight and high hydrophilicity, and the fact that it contains multiple hydroxyl groups, which can ionize and carry a negative charge at physiological pH, *D. magna* may be exposed to this compound through the digestive tract, which may explain its toxicity in this aquatic invertebrate.

To our knowledge, there is only one study that has reported the EC_50_ values of *D. magna* exposed to AT [24], which are very similar to ours (EC_50_ = 50 mg/L). Studies of plant extracts, such as *Pelargonium graveolens*, in which TA is the main component, have shown an LC_50_ (48 h) of 203 mg/L [83]. Other studies have also been conducted with wastewater from the tanning and cork industries, like the one by DeNicola et al. [6], who reported 100% immobilization of *D. magna* individuals at 12.5% of traditional tannery effluents containing up to 0.4 g/L of vegetable tannins. On the other hand, Libralato et al. [84] reported an LC_50_ value, for tannery wastewater, of 26 mg/L for *D. magna* too [23] and provided EC_50_ values ranging from 2.3 to 24.2% dilutions when studying cork-boiling wastewaters containing tannins.

*V. fischeri* is a Gram-negative bacillus widely distributed in marine ecosystems. Its natural bioluminescence is metabolically linked to the cellular respiration AS [76,85] and has been adopted internationally [86] as an indicator of toxicity to marine ecosystems [23].

To the best of our knowledge, this is the first research that provides ecotoxicity data of TA on *V. fischeri.* However, Jochimsen et al. [87] tested tannery wastewaters containing tannins on *V. fischeri*, but they did not provide any EC_50_.

Although is unlikely that TA is able to cross membranes via simple diffusion (as stated before), it seems to be able to disrupt the bacteria envelope and produce cell-oxidative damage [88], which could explain the toxicity effect observed for *V. fischeri*. Samoilova [89] also suggests that the strong oxidative properties of TA override stress responsiveness and alter membrane potential. In this regard, some other studies can be found. For example, TA has significant toxicity in *Staphylococcus aureus* [30], *Escherichia coli* [90,91], *Salmonella enterica* [92], or *Lysteria monocytogenes* [91] strains and *Saccharomyces cerevisiae* yeast [93]. Also, detrimental effects for the normal functioning of *P. aeruginosa* [33] can also be found. However, to the best of our knowledge, there is no available literature regarding the effects of TA on any complex aquatic microbial community.

Our results show that the impact of TA on the river microbial community is significantly lower than in the case of the isolated bacteria *V. fischeri*. This phenomenon has also been observed in other bacteria, such as cyanobacteria [94,95] and bacteria of the *Flavobacteria* genus [96], both of which were present in our samples. All of them exhibited sensitivity to TA when exposed individually, but when they were associated within a community, they appeared to modulate the impact of this compound. 

The river community, as revealed in the genetic study (Supplementary Information Table S2), exhibits a wide diversity of taxa that may display varying sensitivities to this product [97]. So, some bacteria can be directly affected by the disruption of their envelope or intracellular processes. However, those that are more resistant can survive, proliferate, and occupy the niches left by the former. This may explain the limited effect of TA on bacterial growth or even the slight increase observed in comparison to the control (Figure 3), as microbial biomass can be maintained, even though there are changes in biodiversity [98].

On the other hand, microorganisms capable of biodegrading TA have been detected, an effect that can be enhanced when the product is exposed to a diverse community of microorganisms rather than a single species [99,100,101].

Similarly, periphyton, a complex community of aquatic organisms, comprising species of algae, plants, bacteria, fungi, protozoa, and invertebrates, appears to buffer the impact of TA. Periphyton is a basic link in aquatic ecosystems and serves as a valuable indicator of toxicity [102] and water quality [103]. It has been seen that TA can reduce the growth rate and photosynthetic yield of the cyanobacteria *Microcystis aeruginosa* and the unicellular green alga *Desmodesmus armatus* at concentrations ranging from 1 to 20 mg/L, with cyanobacteria being the most sensitive. In fact, the use of these compounds to prevent cyanobacterial and algal growth in freshwater has been proposed [104,105]. However, until now, the effect of TA on river periphyton systems, which integrate the responses of numerous species and their interaction, had not been directly studied. The literature reported that macrophytes heavily colonized by periphyton may have developed a xenobiotic strategy [106] for the release of TA and other polyphenols as a tool in this competition scenario. This strategy may not be enough in highly eutrophicated waters since colonized macrophytes end up reducing their growth until they eventually completely decline [107] and are not able to eliminate the periphyton that covers them, even though it can easily be reached by plants’ freshly released xenobiotics.

According to our results, the architecture of the periphyton itself and the association of several organisms to create these mats with a wide diversity of taxa exhibiting different sensitivities (Supplementary Information Table S2) might provide stronger protection against potentially toxic products, depending on the multispecies competition that is established [108]. 

The density and extracellular polymeric substance (EPS) content can also condition the diffusion properties of periphyton. The EPS improves cell attachment [109] and is a crucial structural property influencing architecture and stability [110], but it also acts as a mechanism of protection for the biofilm microorganisms against toxicants [108,109,110,111]. The protective effect of mucilage has been shown in several studies with chlorine [112] or zinc [113], which causes less damage in biofilms with higher EPS. Recently, it was found that the amine, carbonyl, carboxyl, and hydroxyl groups in EPS were the main functional groups contributing to the interaction of TA with EPS. The existence of EPS reduced the toxicity of TA to algal cells [114].

On the other hand, interactions between periphyton species need to be taken into account when investigating the response to toxic compounds. These interactions can be very complex depending on the taxon composition and the physicochemical conditions of the environment, and they are not well understood in periphyton. It has been reported that interactions with green algae can reverse the inhibitory effects of polyphenols on cyanobacteria, such as *Microcystis aeruginosa* [115]. A similar scenario could be considered in our case, following exposure to TA, among the predominant cyanobacteria in our samples, which are more sensitive to TA [104], and the unicellular algae also present.

Finally, the various taxonomic groups that make up the periphyton exhibit wide metabolic diversity and could potentially play a role in biodegradation. Recent studies have shown that microalgae and cyanobacteria, when associated with bacteria, seem to contribute to the biodegradation of TA [116].

### 3.2. The Effects of TA on Soil Bioindicators and Soil Ecosystems

To the best of our knowledge, this is the first research about TA ecotoxicity in *E. foetida*. This earthworm is defined by the OECD as the main soil model animal and test organism [117]. These earthworms are a vital part of soil ecosystems, mainly due to their ability to degrade organic matter, ensuring their fertility and quality [118]. The fact that they have a very permeable cuticle and a glandular orifice, in addition to a digestive route when they feed in the soil, turns them into a good indicator of soil quality because they are constantly exposed to possible contaminants. However, they seem to be resistant to the effects of TA at the concentrations tested, so it is possible that the effects are not serious enough to cause death. 

Nevertheless, these earthworms could experience some non-observable sublethal effects, such as alterations in their gut microbiota, an effect described in exposure to certain antibiotics, such as oxytetracycline, to which they also appear to be resistant [119]. In fact, TA seems to induce some form of irritation in earthworms, as evidenced by the registration of a product containing this compound to inhibit these terrestrial organisms in 1996. This product was applied by spraying or injecting into the soil to achieve the mentioned effect.

The *Allium cepa* root elongation test has been widely accepted for cytotoxic assessment and genotoxic influence caused by soil, air, and water contamination [120,121]. It has been validated for chemical toxic screening by the International Programme on Chemical Safety (IPCS, WHO) [122] and the United Nations Environment Programme (UNEP) [123]. Our results show, for the first time, that TA presents phytotoxicity against this terrestrial plant, affecting its seed germination. This effect was already registered but in few terrestrial plants, such as legumes of the species *Vigna unguiculata*. Jadhav et al. [50] found inhibitory effects on the seed germination and root and stem growth of this plant at concentrations of TA of 100 mg/L. Seed inhibition by TA was also observed in *Alternanthera tenella* [124]. Further studies are required to clarify the specific mechanisms of action of TA in plant cells.

Finally, the effect of TA on soil microorganisms is stronger than that in river communities, according to some authors who have pointed out that microorganisms present in sediments are much more vulnerable to the stress of potentially toxic elements than aquatic organisms [125,126]. 

Some evidence has been reported in the literature indicating that pine needle tannin extracts, as well as condensed and hydrolysable tannins, exhibit inhibitory activity or induce alterations in nitrogen fixation in soil microorganisms such as Rhizobiales, which are also present in our samples [127,128]. However, this is the first comprehensive study of genetically identified soil communities in this context.

In addition to damaging bacterial cover, other mechanisms of action of TA may explain its antimicrobial effects, especially in communities with high biodiversity, as studied in this research (Figure 2). An important characteristic of tannins that provides a possible explanation for their mode of action on microorganisms is their astringent capacity, which allows them to precipitate proteins by binding to them. This binding could lead to the inhibition of extracellular microbial enzymes [129,130]. These authors suggest that this astringent capacity tends to increase with molecular weight, as is the case with TA. For instance, Mandal et al. [131] demonstrated the inhibition of important resistance-related enzymes, such as beta-lactamases and carbapenemases. It has also been suggested that TA may affect microorganisms by limiting the availability of substrates required for microbial growth, such as via metal deprivation, as tannins can form organo-metallic coordination compounds with some of them. Furthermore, according to the literature, this chelating action increases with the number of oxygen-diphenol groups, which are abundant in TA [132,133]. The observed changes in the metabolic profile may also result from a direct impact on microbial metabolism through the inhibition of oxidative phosphorylation. Other suggested impacts of TA on microorganisms include the inhibition of quorum sensing in bacteria [33] and efflux pumps, as observed in the case of *S. aureus* [134]. 

All this could help explain why this edaphic community seems to be more sensitive than the river community. The literature also reports studies suggesting that soil microorganisms may exhibit somewhat greater sensitivity to potentially toxic compounds compared to aquatic microorganisms [125,126]. This observed effect aligns with the findings of our previous studies involving microbial communities exposed to various compounds, such as plant-based products or extracts [44,135]. However, it is important to consider that several microorganisms have evolved mechanisms to tolerate these high concentrations of tannins [97]. There is also evidence that certain soil bacteria are capable of degrading TA [136,137]. These mechanisms may mitigate its impact on these communities, including the dynamics of replacing sensitive species with more resistant ones.

This is the first study that assesses the ecotoxicity of TA in both aquatic and soil environments using a range of aquatic and terrestrial bioindicators, as well as genetically identified communities. This comprehensive approach provides a more holistic understanding of the potential effects of TA on different ecosystems, which is of utmost interest given the increasing commercialization of this product. Furthermore, the study reveals different sensitivities of various organisms towards TA, presenting valuable information on variable levels of susceptibility among different species.

In this initial study, a wide range of TA concentrations was sought to better evaluate its ecotoxicity. However, it may be valuable to explore, in further research, the effects at lower and more environmentally relevant concentrations to comprehensively evaluate the potential risks associated with even minimal TA exposure. Furthermore, it is essential to consider the impact of environmental factors such as temperature variations, the presence of other contaminants, and ecological interactions, which could significantly influence the actual impact of TA, a consideration that may not be fully captured in a laboratory setting. Finally, while the study primarily focuses on the acute effects of TA exposure on various organisms, it could be beneficial to examine potential long-term chronic effects and the possibility of bioaccumulation in the ecosystem. Considering these aspects could offer an alternative perspective on the overall environmental risk associated with technical assistance.

## 4. Materials and Methods

### 4.1. Chemicals 

All chemicals used in this research, as well as some of their properties, are listed in Table 1.

### 4.2. Daphnia Magna Tests 

To perform the *D. magna* tests (water flea, from Vidrafoc, Spain, ref. DM121219), the OECD 202 (2004) guidelines [138,139] and the standard operational procedures of the Daphtoxkit FTM magna (1996) were followed. Briefly, the planktonic crustaceans were stored at 5 °C until use. *D. magna* eggs were incubated for 72 h at 20–22 °C using 6000 lx light in a TOXKIT model CH-0120D-AC/DC incubator (supplied by ECOTEST, Valencia, Spain). Then, they were fed with spirulina, provided in the kit, 2 h prior to TA exposure.

TA was dissolved into synthetic sterile freshwater (ISO 6341 2012 [131]) to final concentrations of 0.1, 1, 10, and 100 mg/L. This water was also used as a negative control. The pH was adjusted up to 7–7.5 with a 0.1 M NaOH solution. Each concentration was tested with five replicates of five organisms each.

In absolute darkness and for 24 h of exposure at 20–22 °C, daphnids were incubated at the cited concentrations. The organisms that could not swim for 15 s, when gentle agitation was applied, were considered immobile. 

The corresponding EC_50_ and EC_10_ (the effective concentration of TA resulting in 50% and 10% immobilization, respectively) values and their Confidence Intervals (CIs) were obtained from the dose–response curves for the *D. magna* mobility tests using the Xlstat software, Addinsoft (2023), New York, NY, USA (https://www.xlstat.com/essoftware, accessed 27 September 2022).

### 4.3. Vibrio Fischeri Bioluminescence Assay

This experiment was based on the determination of inhibition of the marine bacteria *Vibrio fischeri* (NRRL B-11177) and, therefore, assessed the toxicity of any given contaminant to this marine bioindicator. 

Lyophilizate bacteria were purchased from Macharey-Nagel (ref. 945 006) and stored frozen at −18 °C. The experiments were carried out according to the protocol ISO 11348 [86].

A stock solution of 4000 mg/L of TA was prepared by diluting pure TA in an aqueous solution of 20 g/L NaCl. From this, serial dilutions (0.4, 4, 40, and 400 mg/L) were prepared using the same solvent. The pH of the sample was checked to ensure that it remained within the established parameters (6–8.5) and shaken vigorously for adequate oxygenation. At the same time, the bacterial solution was prepared by adding the culture medium (about 10 mL) provided by the *V. fischeri* kit (purchased from Macharey-Nagel (ref. 945 006) to the freeze-dried vial. All samples and solutions were kept at a moderate temperature (15 ± 1 °C), and four replicates were measured for every sample dilution. 

The experiment consisted of determining the bioluminescence of the bacterial solution after a short resting period (10 min). At this point, aliquots of serial dilutions of TA were then added to an equal volume (1 mL) of the bacterial solution, and the change in bioluminescence was measured after 30 min of exposure. Therefore, the concentrations tested were half of the above-mentioned serial dilutions (0.2, 2, 20, 200, and 2000 mg/L).

The EC_50_ and EC_10_ (the effective concentrations of TA resulting in an inhibition of the bioluminescence of 50% and 10%, respectively) values and their Confidence Intervals (CIs) were obtained from the dose–response curves for *V. fischeri* using the Xlstat software cited before.

### 4.4. Periphyton Communities Assay 

#### 4.4.1. Colonization 

Periphyton communities were collected from the Gállego River, as previously described [44]. Racks were placed 15 cm below the water surface and were removed 15 days later (24 June 2019). At that time, the thickness became 0.75 mm. This meant that the algal biofilm showed communities of similar biomass and physical dimensions [44,140]. Then, the periphyton colonies were taken to the laboratory and prepared for the taxonomical analysis.

#### 4.4.2. Water Samples 

A water sample was collected at the same time and place as the periphyton slides, and the physicochemical parameters of the river were measured (see Supplementary Information Table S1).

#### 4.4.3. Taxonomic Identification 

The identification and counting of algae from the periphyton samples were performed in the laboratory following the Utermöhl technique adapted to inverted microscopy (UNE-EN 15204, 2007 [141]. H_2_O_2_ was applied on the samples to obtain an oxidized and clean frustule suspension. To identify, count, and interpret diatoms in the sample, hydrogen peroxide was mounted on slides with Naphrax© resin (UNE-EN 15204 [133], UNE-EN 13946 [142], and UNE-EN 14407 [143]). 

Diatom cell count and identification were carried out with a Leica light microscope at 1000 magnification, while other algae needed 100, 400, and 1000 magnifications. 

The results are given as density (individuals/mL) (see Supplementary Information Table S2).

#### 4.4.4. Dose and Time Response Curves in Flow-Through Artificial Channels 

The toxicity tests were conducted in methacrylate flow-through pipes that were connected to various water reservoirs. Every channel received 0.113 m^3^/h of water from a closed water circuit that was fed by a series of motors connected to various reservoirs. A thermostatic bath kept the water at a constant 23 °C. The volume of each reservoir was the same (4 L).

The slides colonized with periphyton recently brought from the river were laid horizontally in an acclimatization channel at 23 °C before the ecotoxicology studies began. The slides were then positioned horizontally at the base of six fake flow-through tubes (mesocosms). 

The light was delivered via lamps (Blau aquaristic, T5HO, 39 w/10,000 K, 80 μmol photon/s·m^2^ on the channel surface) designed specifically to produce algae, which had a light spectrum similar to that of sunshine. This allowed periphyton communities to carry out photosynthesis in a manner that was representative of actual environmental conditions. The periphyton organisms were treated with TA at doses of 0.1, 1, 10, 100, and 1000 mg/L in a buffer solution (MOPS, 0.01 M) that had been pH-adjusted to 7.5, using either NaOH or HCl. As a negative control, one channel with MOPS but no TA was utilized. To ensure steadiness during the experiment, the temperature was checked on a regular basis. 

Similarly to that previously described by Pino-Otín et al. [44], the effect of TA on the photosynthetic efficiency of the periphyton was assessed by measuring the photosynthetic yield, which represents the effectiveness of the photochemical energy conversion process [144], in triplicate after 1 and 2 h of exposure. 

Since the structure of each community can differ and have an impact on the toxicity of the chemicals, a control sample was taken at time 0 for each measurement. 

The EC_50_ and EC_10_ values (the effective concentrations of TA resulting in 50% and 10%, respectively, of photosynthetic yield) and their Confidence Intervals (CIs) were obtained from the dose–response curves for periphyton using the Xlstat software cited before.

### 4.5. Water and Soil Microorganism Tests 

#### 4.5.1. Water Samples 

In accordance with established protocols (ISO 19458:2006 [145]), water samples were taken from the Gállego River in Zaragoza, Spain, at the same time as the periphyton samples (Table S3).

Microorganisms were isolated for genetic analysis from 5 L of river water that had been filtered using a 0.22 mm cellulose nitrate filter (Sartorius), resuspended in a sterile Falcon tube with 50 mL of phosphate-buffered saline (PBS), sterilized, and centrifuged for 10 min at 5000× *g*. Before sequencing, the pellet was kept at −80 °C, while the supernatant was discarded.

1 L of river water was filtered to remove debris using a 70 mm nylon sieve (BD Falcon) and then stored at 4 °C in the dark until use in the ecotoxicity experiments.

#### 4.5.2. Soil Samples 

The soil was taken from a crop field devoid of pesticides or other contaminants (CITA, Zaragoza, NE Spain). The composition of the soil was examined (CITA Soil and Irrigation Unit, Table S4).

For the genetic study, we followed previous procedures [44]. Briefly, 20 g of soil was mixed with 100 mL of sterile water for 30 min before being allowed to stand for an additional hour. The sample was then separated into 10 mL Falcon tubes and centrifuged at 1000× *g* for 10 min after being subjected to a 1 min sonication. The supernatant was sterilely obtained. The soil microorganisms were recovered by filtering the supernatant using a vacuum Büchner flask and a 0.22 mm Sartorius cellulose nitrate filter. After carefully washing the filter material in sterile PBS, the sample was centrifuged at 5000× *g* for 10 min. A dropper was used to extract the supernatant, and the pellets were then kept at −80 °C until sequencing.

Using a 2 mm sieve, 10 g of soil was filtered (Becton Dickinson, Zaragoza, Spain) before the ecotoxicity tests. The 95 mL of sterile water was added to the 10 g of pre-sieved soil, and the sample was stirred in an Erlenmeyer flask for 30 min before standing for an hour. The supernatant was then collected under sterile circumstances and 10 mL of the Erlenmeyer flask’s top was centrifuged at 1000× *g* for 10 min. There were five iterations of this cycle. The resulting whole supernatant was filtered to remove suspended soil debris with a 70 mm nylon sieve (Becton Dickinson, Spain).

#### 4.5.3. Community-Level Physiological Profiling (CLPP) of Water and Soil Samples 

TA’s effects on the metabolism of microbial communities from water and soil and, specifically, alterations in the use of 31 distinct carbon sources, as previously described, were evaluated using the Biolog^®^ EcoPlates (Tiselab S.L., Barcelona, Spain) [44,146]. 

To determine the toxicity of TA on the communities of microorganisms from soil and water, respectively, the following dilutions of TA were prepared for the ecotoxicity test: 0.2, 2, 20, and 200 mg/L in a final volume of 150 mL in the wells of a Biolog^®^ EcoPlates with prefiltered river water, or the supernatant obtained from the soil sample. The dilutions’ ultimate pH ranged from 6 to 7. Three replicates of each concentration were tested. All manipulations were carried out in a flow chamber in a sterile environment. The plates underwent a 6-day sterile incubation period in the darkness at 25 °C.

With the help of Gen5TM data analysis software and a Synergy H1 Microplate Reader (BIO-TEK, Winooski, VT, USA), the OD (wavelength 590 nm) of each well was measured immediately upon inoculation and once per day for 144 h. Thus, the decrease in tetrazolium violet redox was used to measure the rate of utilization of the carbon sources. The average well color development (AWCD) was calculated for every one of the 3 replicates of the 96-well plate, as previously described by [147], as follows in Equation (1):

AWCD = S(AbsW − AbsC)/31(1)

where AbsW is the absorbance (or optical density) of each well with the carbon source and AbsC is the absorbance of the control well without it.

To study the different consumption levels of metabolites by the water and soil microorganisms, these were grouped as Weber et al. [148] previously described. After that, the AWCD was determined for each group according to Equation (1).

AWCD values are given as the average of the replicates ± standard deviation.

#### 4.5.4. Genetic Sequencing of River and Soil Microorganisms 

Through genetic sequencing, the taxonomic composition and the predominate taxa of these microbial communities were examined to better understand the impact of this natural compound on their growth and metabolism.

The Genomics Unit Cantoblanco, Science Park, performed the genetic sequencing of water and soil microbes (Madrid, Spain). After being previously homogenized in PBS, the samples’ bacterial genomic DNA was extracted using G-spin columns from 200 mL aliquots (INTRON Biotechnology, Seongnam, Kyonggi-do, Repulic of Korea). DNA concentration was assessed using Thermo Fischer’s Quant-IT PicoGreen reagent. As previously stated, DNA samples were utilized to amplify the V3-V4 region of the 16S ribosomal RNA (rRNA) gene [44,149,150]. 

Individual amplicon libraries were analyzed on an Agilent Bioanalyzer 2100, and the concentration was determined using real-time PCR (Kapa Biosystems). DNA samples were sequenced using an Illumina MiSeq instrument in accordance with a 2 × 300 procedure. Utilizing current Base Space applications, reads were quality-filtered using Illumina standard values, demultiplexed, and fastq files were mapped to the GreenGenes database (16S Metagenomics, Illumina, San Diego, USA).

In the run, all 145,498 reads for soil microorganisms and all 85,525 reads for water microorganisms passed the quality filtering with a perfect score.

### 4.6. Eisenia Foetida Assays 

We purchased adult *Eisenia foetida* organisms from Todo Verde’s composters (Zaragoza, Spain). Earthworms were conditioned in a sphagnum peat substrate from the Spanish Flowers Company (Zaragoza, Spain) for 15 days prior to testing. They were maintained in a stable environment at 18–25 °C, a pH of 7.8–8, and a humidity level of 80–85%.

Adult earthworms that were older than 60 days were selected. They all had a clitellum and similar size, and weighed between 300 and 600 mg. The OECD 207 (1984) [117] protocol was followed for the toxicity tests in a similar manner to that previously reported [44,78].

Commercial black peat (Verdecora vivarium, Spain), kaolinic clay, and quartzitic sand were combined in the following proportions to create the artificial soil, which was created in accordance with OECD 207 standard: 7:2:1 [44,78]. Deionized water was used to alter the substrate’s moisture content in an amount equal to 35 to 45% of the dry weight of the soil. An amount of 600 mg of this synthetic soil was placed in 1 L capacity polypropylene containers with perforated lids to prevent moisture loss.

Each container included ten earthworms, and 120 mL of the TA dilutions 0.2, 2, 20, 200, and 2000 mg/L was added. The same method was used to create negative controls without TA. Each concentration was examined three times. A regulated environment of 20 ± 2 °C, 80–85% relative humidity, and 400–800 lx of light was used to maintain the containers. Earthworm mortality was assessed 14 days after treatment.

The EC_50_ and EC_10_ (the effective concentrations of TA resulting in 50% and 10%, respectively, of worm survival) values and their Confidence Intervals (CIs) were obtained from the dose–response curves for *E. faetida* using the Xlstat software cited before.

### 4.7. Allium cepa Assay 

*Allium cepa* (variety Stuttgarter Riesen de 14/21) bulbs were purchased from Fitoagrícola Company (Spain) and kept in a dry environment between 10 and 20 °C in the dark until use to prevent the growth of fungi. Before the test, the young bulbs were peeled. Damage to the root ring was prevented with this operation.

The protocol developed by Fiskesjö (1993) [120] was followed to conduct *A. cepa* acute toxicity tests, consisting of the measurement of root elongation after 72 h of exposure to the test substance [44]. Because it has a suitable amount of calcium and magnesium ions for the plant to grow properly [44,151], mineral water from Aguas de San Martín de Veri S.A. (https://www.veri.es/es/el-producto, accessed 18 September 2022) from Spain was used as the growing medium for the bulbs, which were put in 15 mL tubes. Twelve replicates of each concentration (0.2, 2, 20, 100, and 500 mg/L) were used in ecotoxicological studies. The only component of the negative control was water. The bulbs were grown for 72 h at 25 °C in a dark room. Every 24 h, the test solutions were renewed.

The EC_50_ and EC_10_ (the effective concentrations of TA resulting in 50% and 10%, respectively, of root growth) values and their Confidence Intervals (CIs) were obtained from the dose–response curves for *A. cepa* using the Xlstat software cited before.

### 4.8. Statistics and Graphical Representation 

Using the Xlstat software cited before, logit logistic regression was applied to create the dose–response curves for *D. magna* mobility, *E. foetida* survival, *A. cepa* root elongation, and periphyton communities’ photosynthetic yield in order to determine the appropriate EC_50_ and EC_10_ values [44]. A chi-squared test was used to statistically evaluate dose–response models. Also, with the Xlstat software cited before, Student’s *t*-test on two independent samples and the variance association between the AWCD values of the three replicates were calculated to determine significance.

## 5. Conclusions

Our results show, for the first time, that TA can have an impact on aquatic organisms, especially for isolated nekton bacteria such as *V. fischeri* or invertebrates like *D. magna*. However, the river microbial communities of nekton and periphyton seem to be able to protect themselves or balance the effects that this product can produce on individual organisms, even biodegrading it. Earthworms also seem to have mechanisms to, at least, not suffer lethal effects when exposed to high concentrations of TA. However, this compound can present phytotoxicity in plants of economic interest, such as *A. cepa*, and produce a significant impact on both the growth and metabolic profiles of edaphic microbial communities. These microorganisms are the basis of trophic chains, so their alteration could have a significant impact on soil quality, especially at high doses that are not currently present in the environment. However, at the present time, this product is already being marketed for multiple commercial uses and its consumption is likely to increase in the coming years, so this study can shed light on the possible toxicity effects that it can cause in aquatic and terrestrial environments. Likewise, these results emphasize that products of natural origin must be studied from the point of view of their ecotoxicity and be subjected to the same supervision as synthetic ones so that they can become a more ecofriendly alternative in an industry that is increasingly committed to sustainability.

## Figures and Tables

**Table 1:** Name of the chemical, CAS number, purity, and company where all the chemicals used in this research were purchased from.

**Name of the Chemical**	**CAS Number**	**Purity**	**Company**
Tannic acid	1401-55-4	ACS Reagent (>95%)	Sigma-aldrich (Burlington, VT, USA)
NaOH	1310-73-2	98%	PanReac (Barcelona, Spain)
HCl	7647-01-0	37%	Fisher Chemical (Pittsburgh, PA, USA)
PBS (phosphate-buffered solution)	-	-	Sigmaaldrich (Burlington, VT, USA)
MOPS (4-Morpholinepropanesulfonic acid, 3-(N-Morpholino) propanesulfonic acid)	1132-61-2	≥99.5%	Sigma-aldrich (Burlington, VT, USA)
Ethanol	64-17-5	≥99.9%	Supelco by Sigma-aldrich (Burlington, VT, USA)

## Data Availability

No new data were created.

## Supplementary Materials

The following supporting information can be downloaded at: https://www.mdpi.com/article/10.3390/plants12234041/s1, Figure S1: Dose–response curve for periphyton test after 2 h exposure to tannic acid (TA). Photosynthetic values are given as a ratio with respect to the control, and each dose was measured in triplicate. Red line is the model, and dashed lines are the inferior and superior confidence limits (95%); Figure S2: Dose–response curve for *Eisenia foetida* test after 14-day exposure to tannic acid. Curves are the average of 3 replicates. Red line is the model; Table S1: Physico-chemical parameters of water and soil samples; Table S2: Diatom cell count and identification of periphyton communities; Table S3: Water samples’ taxonomy; Table S4: Soil samples’ taxonomy.
