# Supplementary material for: Ecotoxicological Study of Tannic Acid on Soil and Water Non-Target Indicators and Its Impact on Fluvial and Edaphic Communities
Source: Plants (Basel). 2023 Nov 30;12(23):4041. doi: 10.3390/plants12234041 (PMC10708037; doi:10.3390/plants12234041)
Supplement: Supplementary file 1 [file plants-12-04041-s001.zip › plants-2705566-supplementary.pdf]

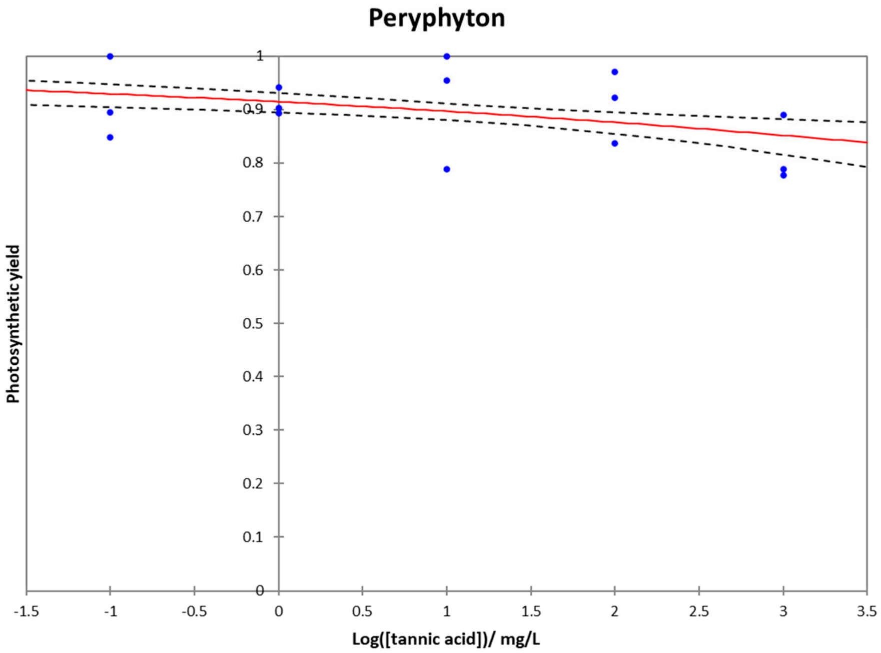

Figure S1. Dose-response curve for periphyton test after 2 hour-exposure to tannic acid (TA). Photosynthetic values are given as a ratio with respect the control, and each dose was measured in triplicate. Red line is the model and dashed lines are the inferior and superior confidence limits (95%).

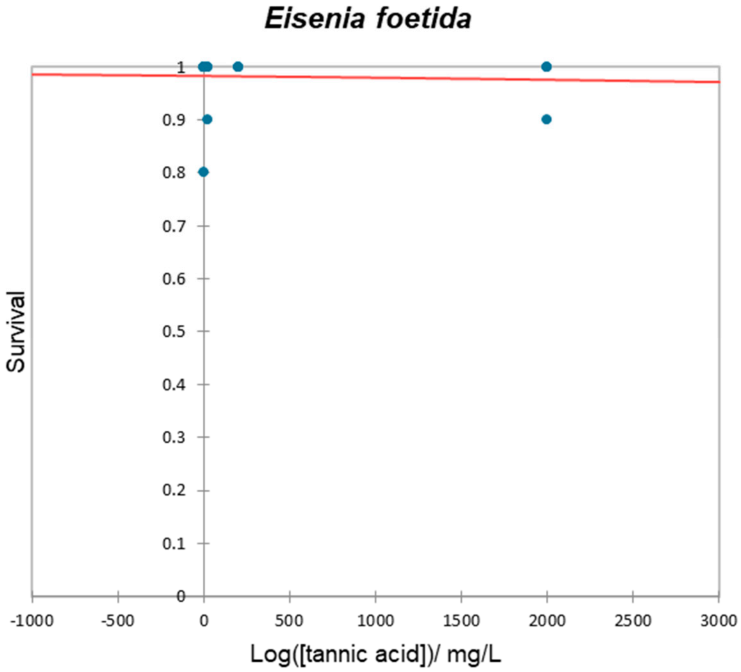

Figure S2. Dose-response curve for *Eisenia foetida* test after 14 day-exposure to TA. Curves are the average of 3 replicates. Red line is the model.

Table S1. Physico-chemical parameters of water and soil samples.

River		Soil	
Parameter	Value	Parameter	Value
Conductivity (mS/cm)	2317.0	Clay content (%)	11.7
Total suspended solids (mg/L)	5.1	Sand content (%)	50.5
Organic matter (mg/L)	3.0	Silt content (%)	37.8
Total dissolved solids(mg/L)	1586.4	K (mg/L)	109.3
Carbonates (mg/L)	0.0	P Olsen (mg/Kg)	10.4
Bicarbonates (mg/L)	249.4	Ca meq 100	23.8
Fluorides (mg/L)	0.1	$EC_{1:5}$ (dS/m)	0.6
Chlorides (mg/L)	475.2	Total nitrogen (%)	0.1
Nitrites (mg/L)	0.0	$CaCO_3$ (%)	42.3
Bromides (mg/L)	0.5	pH	8.1
Nitrates (mg/L)	14.5		
Phosphates (mg/L)	0.0		
Sulphates (mg/L)	358.8		
Total alkalinity (mg/L)	294.4		
Total organic carbon (mg/L)	3.0		
Total nitrogen (mg/L)	3.8		
pH	7.9		

Table S2. Diatom cell count and identification of periphyton communities.

Taxonomic identification		Density (individuals/mL)
**Non diatom**		
Cyanobacteria	*Chroococcus* sp.	2
	Leptolyngbya sp.	53
	Oscillatoria sp.	16
	Phormidium sp.	29
	Pleurocapsa sp.	5
	Pseudanabaena sp.	2
Rhodophyta	*Bangia* sp.	52
Chlorophyceae	*Draparnaldia* sp.	15
	Oedogonium sp.	102
	Pediastrum boryanum	4
	Scenedesmus spp.	45
Ulvophyceae	*Cladophora* sp.	15
	Gongrosira sp.	5
	Ulothrix sp.	247
Conjugatophyceae	*Spirogyra* sp.	4
**Diatom**		
Bacillariophyceae	*Achnanthidium eutrophilum* (Lange-Bertalot)Lange-Bertalot	62
	Achnanthidium minutissimum (Kütz.) Czarnecki f. *anormale*	2
	Achnanthidium minutissimum (Kützing) Czarnecki	310
	Achnanthidium pyrenaicum (Hustedt) Kobayasi	493
	Achnanthidium straubianum (Lange-Bertalot)Lange-Bertalot	4
	Amphora copulata (Kütz) Schoeman & Archibald	6
	Amphora inariensis Krammer	2
	Amphora pediculus (Kützing) Grunow	81
	Cocconeis euglypta Ehrenberg	4
	Cocconeis euglyptoides (Geitler) Lange-Bertalot	2
	Cocconeis pediculus Ehrenberg	10
	Cyclotella meneghiniana Kützing	10
	Diatoma moniliformis (Kützing) Williams ssp. *moniliformis*	2
	Encyonema silesiacum (Bleisch in Rabh.) D.G. Mann	2
	Eunotia sinuosa Hustedt	2
	Gomphonema saprophilum (Lange-Bertalot & Reichardt) Abarca Jahn Zimmermann & Enke	33
	Halamphora veneta (Kützing) Levkov	19
	Navicula antonii Lange-Bertalot	6
	Navicula cryptotenella Lange-Bertalot	4
	Nitzschia amphibia Grunow	27
	Nitzschia aurariae Cholnoky	2
	Nitzschia dissipata (Kützing) Grunow ssp. *dissipata*	10
	Nitzschia fonticola Grunow in Cleve et Möller	19
	Nitzschia frustulum (Kützing) Grunow	6
	Nitzschia inconspicua Grunow	121
	Nitzschia microcephala Grunow in Cleve & Moller	8
	Nitzschia palea (Kützing) W.Smith	2
	Planothidium frequentissimum (Lange-Bertalot) Lange-Bertalot	85
	Planothidium lanceolatum (Brébisson ex Kützing) Lange-Bertalot	12
	Rhoicosphenia abbreviata (C.Agardh) Lange-Bertalot	2
	Staurosira venter (Ehrenberg) Cleve & Moeller	62
	Surirella brebissonii var. *kuetzingii* Krammer et Lange-Bertalot	2

Table S3. Water samples taxonomy.

Phylum	%	Class	%	Order	%	Family	%	Genus	%	Species	%
Cyanobacteria	40.2	Oscillatoriophycideae	30.1	Chroococcales	30.1	Unclassified	29.1				
		Nostocophycideae	7.5	Stigonematales	4.9	Rivulariaceae	4.9	*Calothrix*	4.9	*Parietina*	0.4
										Other *Calothrix*	0.0
										Unclassified *Calothrix*	4.5
				Nostocales	2.5	Nostocaceae	2.5	Other *Nostocaceae*	0.0		
								Unclasiffied	2.5		
						Other Nostocaceae	0.0				
				Other	0.1						
		Other	2.6								
Proteobacteria	36.1	Betaproteobacteria	17.4	Burkholderiales	16.0	Comamonadaceae	15.7	*Hydrogenophaga*	3.3	*defluvii*	1.0
										pseudoflava	0.3
										Other	0.2
										Unclassified	1.7
								Limnohabitans	7.2	*parvus*	2.4
										Other	0.4
										Unclassified	4.4
								Paucibacter	1.4	Unclassified	1.4
								Other *Comamonadaceae*	2.5		

								Unclassified *Comamonadaceae*	1.3		
						Other Burkholderiales	0.4				
				Other Betaproteobacteria	1.4						
		Alphaproteobacteria	8.4	Rhodobacterales	5.0	Rhodobacteraceae	4.9	*Rhodobacter*	4.1	*ovatus*	0.6
										Other	0.2
										Unclassified	3.3
								Paracoccus	0.3	*sulforoxidans*	0.1
										Other	0.0
										Unclassified	0.2
								Other	0.5		
						Other	0.1				
				Sphingomonadales	1.4	Sphingomonadaceae	1.3	*Novosphingobium*	0.7	*lentum*	0.1
										Other	0.1
										Unclassified	0.5
								Sphingomonas	0.3	*hunanensis*	0.1
										Other	0.1
										Unclassified	0.1
								Zymomonas	0.1	Unclassified	0.1
								Other	0.0	Other	0.5

								Unclassified	0.1	Unclassified	0.1
						Erythrobacteraceae	0.1	*Erythromicrobium*	0.1	*ramosum*	0.1
										Unclassified	0.0
								Erythrobacter	0.0	*aquimaris*	0.0
								Other	0.0	Other	0.0
						Other	0.0				
				Rhizobiales	1.1	Rhizobiaceae	0.4	*Agrobacterium*	0.2	*tumefaciens*	0.1
										albertimagni	0.0
										viscosum	0.0
										Other	0.0
								Rhizobium	0.0	*alamii*	0.0
										pisi	0.0
										Other	0.0
								Canditatus liberibacter	0.0	*solanacearum*	0.0
										Other	0.0
										Unclassified	0.0
								Other	0.1		
				Other	0.9						
		Gammaproteobacteria	7.1	Alteromonadales	2.9	Chromatiaceae	2.2	*Rheinheimera*	2.0	*texasensis*	0.6
										chironomi	0.3
										Other	0.1
										Unclassified	1.0

								Alishewanella	0.2	Unclassified	2.0
								Other	0.0		
						Alteromonadaceae	0.4	*Cellvibrio*	0.3	*ostraviensis*	0.1
										Other	0.0
										Unclassified	0.2
								Candidatus endobugula	0.1	*glebosa*	0.0
										Other	0.0
										Unclassified	0.0
								Glaciecola	0.0	*nitratireducens*	0.0
								Other	0.1		
						Shewanellaceae	0.2	*Shewanella*	0.2	*upenei*	0.0
										oneidensis	0.0
										Other	0.0
										Unclassified	0.1
						Other	0.1				
				Pseudomonadales	1.0	Pseudomonadaceae	0.7	*Pseudomonas*	0.6	*anguilliseptica*	0.0
										plecogossicida	0.1
										alcaligenes	0.1
										Other	0.1
										Unclassified	0.2
								Azomonas	0.1	*macrocytogenes*	0.0

										Unclassified	0.1
								Other	0.0		
						Moraxellaceae	0.3	*Alkanindiges*	0.1	Unclassified	0.1
								Acinetobacter	0.2	*tjernbergiae*	0.0
										antiviralis	0.0
										gerneri	0.0
										Other	0.1
										Unclassified	0.1
								Psychrobacter	0.0	Unclassified	0.0
										Other	0.0
								Other	0.0		
						Other	0.0				
				Enterobacteriales	0.6	Enterobacteriaceae	0.6	*Plseiomonas*	0.5	Unclassified	0.5
								Yersinia	0.1	*massiliensis*	0.0
										unclassified	0.0
										Other	0.0
								Other	0.1		
				Xanthomonadales	0.7	Xanthomonadaceae	0.5	*Arenimonas*	0.1	*malthae*	0.1
										Unclassified	0.0
										Other	0.0
								Thermomonas	0.1	*brevis*	0.0
										dokdonensis	0.0

										Unclassified	0.0
								Luteimonas	0.0	*terricola*	0.0
										aquatica	0.0
										Other	0.0
								Aquimonas	0.0	*voraii*	0.0
										Unclassified	0.0
								Xanthomonas	0.0	Other	0.0
										Unclassified	0.0
								Other	0.0		
								Unclassified	0.1		
						Sinobacteraceae	0.2	*Steroidobacter*	0.2	*denitrificans*	0.2
								hydrocarboniphaga	0.0	*daquingensis*	0.0
										Unclassified	0.0
				Oceanospirillales	0.5	Oceanospirillaceae	0.2	*Marinomonas*	0.1	*basaltis*	0.0
										brasiliensis	0.0
										Other	0.0
										Unclassified	0.0
								Marinospirillum	0.0	Unclassified	0.0
								Marinobacterium	0.0	*sediminicola*	0.0
								Amphritea	0.0	*atlantica*	0.0
								Other	0.0		

								Unclassified	0.0		
						Halomonadaceae	0.3	*Halomonas*	0.2	Unclassified	0.2
										Other	0.0
								Kushneria	0.1	*indalinina*	0.0
										Unclassified	0.0
										Other	0.0
								Other	0.0		
						Other	0.1				
				Other	1.4						
		Other Proteobacteria	3.2								
Bacteroidetes	11.7	Flavobacteriia	5.3	Flavobacteriales	5.3	Flavobacteriaceae	4.4	*Flavobacterium*	3.4	Unclassified	2.6
										Other	0.8
								Tenacibaculum	0.4	*litopenaei*	0.2
										Unclassified	0.2
										Other	0.0
								Polaribacter	0.3	*butkevichii*	0.2
										Other	0.0
										Unclassified	0.2
								Other	0.4		
						Cryomorphaceae	0.9	*Fluviicola*	0.9	Unclassified	0.9
						Other	0.0				
		Sphingobacteriia	5.9	Sphingobacteriales	5.3	Flexibacteraceae	2.3	*Arcicella*	1.2	Unclassified	1.2

								Runella	0.4	*limonsa*	0.4
										Other	0.0
								Hymenobacter	0.4	Unclassified	0.3
										Other	0.1
								Emticicia	0.3	*oligotrophica*	0.2
										Unclassified	0.1
										Other	0.0
								Other	0.1		
						Chitinophagaceae	1.5	*Chitinophaga*	0.1	*soli*	0.1
										Other	0.0
								Segetibacter	0.3	*aerophilus*	0.2
										Other	0.0
								Other	0.1		
								Unclassified	1.0		
						Saprospiraceae	0.8	*Lewinella*	0.6	*marina*	0.4
										Unclassified	0.2
										Other	0.0
								Haliscomenobacter	0.1	*hydrossis*	0.0
										Unclassified	0.1
								Other	0.0		
						Sphingobacteriaceae	0.4	*Pedobacter*	0.2	*kwangyangensis*	0.1
										Unclassified	0.2

										Other	0.0
								Olivibacter	0.1	Unclassified	0.1
										Other	0.0
								Sphingobacterium	0.0	*bambusae*	0.0
										shayense	0.0
								Unclassified	0.0		
								Other	0.0		
						Other	0.4				
		Other	0.5								
Other	11.6										

Table S4. Soil samples taxonomy.

Phylum	%	Class	%	Order	%	Family	%	Genus	%	Species	%
Actinobacteria	49.5	Actinobacteria	33.3	Actinomycetales	30.1	Nocardioidaceae	7.6	*Nocardioides*	3.9	*isalandensis*	2.4
										Unclassified	1.5
										Other	0.02
								Kribbella	1.7	*ginsengisoli*	0.8
										koreensis	0.4
										Other	0.1
										Unclassified	0.4
								Aeromicrobium	1.5	*ponti*	0.6
										ginsengisoli	0.5
										Other	0.3
										Unclassified	0.2
						Psudonocardiaceae	5.5	*Saccharopolyspora*	4.1	Other	0.3
										Unclassified	3.8
								Pseudonocardia	0.7	*hydrocarbonoxydans*	0.1
										kongjuensis	0.1
										Other	0.2
										Unclassified	0.4
								Other	0.7		
						Actinosynnemataceae	2.9	*Lentzea*	0.8	*waywayandensis*	0.7
										Other	0.1
								Kutzneria	0.6	Unclassified	0.6
								Crosiella	0.2	Unclassified	0.2
								Actinokineospora	0.2	*diospyrosa*	0.1
										inagensis	0.1
										Other	0.01
								Saccharothrix	0.2	*longispora*	0.1
										yanglingensis	0.1
										Other	0.00
										Unclassified	0.01
								Other	0.1		
								Unclassified	0.6		
						Other	14.4				
						Unclassified	2.7				
				Other	0.2						
		Thermoleophilia	12.6	Solirubrobacterales	12.6	Conexibacteraceae	6.5	*Conexibacter*	6.5	Other	0.2
										Unclassified	6.3
						Solirubrobacteraceae	5.6	*Solirubrobacter*	5.6	*soli*	3.1
										Unclassified	2.6

						Other	0.5				
		Other	3.6								
Proteobacteria	20.4	Alphaproteobacteria	11.7	Sphingomonadales	4.1	Sphingomonadaceae	3.9	*Kaistobacter*	2.5	*terrae*	0.6
										Unclassified	1.9
								Sphingomonas	0.9	*oligophenolica*	0.2
										Other	0.3
										Unclassified	0.4
								Other	0.2		
								Unclassified	0.3		
						Other	0.2				
				Rhizobiales	4.8	Hyphomicrobiaceae	1.2	*Rhodoplanes*	0.6	*cryptolactis*	0.01
										Other	0.03
										Unclassified	0.5
								Devosisa	0.4	*ginsengisoli*	0.03
										Other	0.1
										Unclassified	0.2
								Hyphomicrobium	0.2	*vulgare*	0.01
										aestuarii	0.03
										Unclassified	0.1
								Other	0.04		
						Bradyrhizobiaceae	0.7	*Balneimonas*	0.6	Unclassified	0.6
								Bradyrhizobium	0.1	*pachyrhizi*	0.01
										Other	0.00
										Unclassified	0.1
								Other	0.04		
						Methylobacteriaceae	0.7	*Methylobacterium*	0.7	*goesingense*	0.5
										Other	0.1
										Unclassified	0.1
						Rhizobiaceae	0.5	*Sinorrhizobium*	0.2	*meliloti*	0.1
										fredii	0.1
										Other	0.02
										Unclassified	0.1
								Agrobacterium	0.1	*albertimagni*	0.01
										Other	0.01
										Unclassified	0.01
								Kaistia	0.1	*granuli*	0.05
										Other	0.00
								Shinella	0.1	*fucsa*	0.04
										Other	0.00
										Unclassified	0.01
									0.1	*africanus*	0.01

								Candidatus liberibacter		Other	0.00
										Unclassified	0.02
								Other	0.1		
						Phyllobacteriaceae	0.5	*Mesorhizobium*	0.3	*camelthorni*	0.1
										alhagi	0.04
										Other	0.01
										Unclassified	0.1
								Phyllobacterium	0.1	*ifriquiyense*	0.02
										bourgognense	0.01
										Other	0.01
										Unclassified	0.04
								Aminobacter	0.1	*amonivorans*	0.03
										aganoensis	0.01
										Other	0.00
										Unclassified	0.04
								Chelativorans	0.02	Unclassified	0.02
								Other	0.04		
						Xanthobacteraceae	0.4	*Ancylobacter*	0.2	*aquaticus*	0.04
										Other	0.00
										Unclassified	0.1
								Labrys	0.1	Other	0.01
										Unclassified	0.1
								Blastochloris	0.1	*gulmargensis*	0.1
										Other	0.00
								Azothizobium	0.03	Unclassified	0.03
								Xanthobacter	0.03	*polyaromaticivorans*	0.02
										viscosus	0.01
										Other	0.00
								Other	0.01		
						Rhodobiaceae	0.2	*Afifella*	0.2	Unclassified	0.2
						Beijerinckiaceae	0.2	*Chelatococcus*	0.2	*daeguensis*	0.2
								Other	0.01		
						Other	0.4				
				Rhodospirillales	1.5	Rhodospirillaceae	1.1	*Rhodovibrio*	0.4	*sodomensis*	0.4
								Azospirillum	0.3	*palatum*	0.04
										Other	0.00
										Unclassified	0.2
								Skermanella	0.2	*aerolata*	0.1
										Unclassified	0.1
								Oleomonas	0.1	*sagaranensis*	0.03
										Unclassified	0.04

								Roseospira	0.1	*visakhapatnamensis*	0.01
										Other	0.00
										Unclassified	0.04
								Rhodospirillum	0.1	Unclassified	0.05
								Other	0.1		
						Acetobacteraceae	0.1	*Roseomonas*	0.05	*massiliensis*	0.01
										terpenica	0.00
										aquatica	0.00
										Other	0.00
										Unclassified	0.03
								Gluconobacter	0.02	*morbifer*	0.01
										Other	0.00
										Unclassified	0.01
								Acidisoma	0.01	*tundrae*	0.01
								Other	0.00	Other	0.00
								Unclassified	0.01		
						Unclassified	0.3				
				Other	1.3						
		Deltaproteobacteria	4.3	Myxococcales	2.6	Cystobacteraceae	1.2	*Cystobacter*	1.2	Other	0.1
										Unclassified	1.1
						Polyangiaceae	0.7	*Chondromyces*	0.7	*pediculatus*	0.6
										Other	0.03
								Other	0.02		
						Myxococcaceae	0.3	*Corallococcus*	0.2	*exiguus*	0.2
								Myxococcus	0.1	*fulvus*	0.04
										xanthus	0.03
										Other	0.01
								Anaeromyxobacter	0.04	Unclassified	0.04
								Other	0.01		
						Haliangiaceae	0.2	*Haliangium*	0.2	Other	0.01
										Unclassified	0.2
						Nannocystaceae	0.1	*Nannocystis*	0.1	Other	0.01
										Unclassified	0.1
						Other	0.03				
				Desulfovibrionales	0.7	Desulfovibrionaceae	0.6	*Desulfovibrio*	0.6	*butyratiphilus*	0.1
										oryzae	0.1
										Other	0.04
										Unclassified	0.4
						Desulfonatronumaceae	0.1	*Desulfonatronum*	0.1	*thiosulfatophilum*	0.1
						Other	0.01				
				Desulfuromonadales	0.4	Geobacteraceae	0.2	*Geobacter*	0.2	*pickeringii*	0.1

										toluenoxydans	0.05
										Unclassified	0.04
						Desulfuromonadaceae	0.02	*Desulfuromusa*	0.02	Other	0.00
										Unclassified	0.02
								Other	0.00		
						Unclassified	0.2				
				Syntrophobacterales	0.2	Syntrophaceae	0.2	*Desulfomonile*	0.2	*tiedjei*	0.2
								Other	0.00		
						Desulfobacteraceae	0.03				
						Syntrophobacteraceae	0.02	*Desulfacinum*	0.01	*subterraneum*	0.01
								Syntrophobacter	0.00	*wolinii*	0.00
								Unclassified	0.01		
						Other	0.02				
				Other	0.4						
		Gammaproteobacteria	2.2	Xanthomonadales	0.9	Xanthomonadaceae	0.5	*Dokdonella*	0.4	*fugitiva*	0.4
								Lysobacter	0.03	*yangpyeogensis*	0.01
										soli	0.01
										niabensis	0.00
										Other	0.00
										Unclassified	0.00
								Other	0.1		
						Sinobacteraceae	0.4	*Steroidobacter*	0.4	*denitrificans*	0.2
										Unclassified	0.2
								Hydrocarboniphaga	0.02	*daqingensis*	0.02
				Chromatiales	0.6	Chromatiaceae	0.4	*Thiocapsa*	0.2	Unclassified	0.2
								Other	0.03		
								Unclassified	0.2		
						Ectothiorhodospiraceae	0.1	*Methylonatrum*	0.02	*kenyense*	0.02
								Halorhodospira	0.02	*halochloris*	0.02
								Ectothiorhodospira	0.01	*haloalkaliphilia*	0.01
								Other	0.00		
								Unclassified	0.05		
						Other	0.04				
				Other	0.4						
				Unclassified	0.4						
		Other	2.2								
Firmicutes	13.3	Clostridia	8.1	Clostridiales	4.0	Clostridiaceae	1.3	*Clostridium*	1.1	Other	0.1
										Unclassified	0.9
								Peptoniphilus	0.1	*coxii*	0.03
										Other	0.01
										Unclassified	0.1

								Other	0.1		
						Lachnospiraceae	0.7	*Blautia*	0.4	*coccoides*	0.1
										hansenii	0.04
										Other	0.04
										Unlassified	0.2
								Coprococcus	0.1	Unclassified	0.1
								Roseburia	0.1	*faecis*	0.01
										Unclassified	0.1
								Lachnospira	0.1	*pectinoschiza*	0.04
										Unclassified	0.01
								Ruminococcus	0.04	Unclassified	0.04
								Other	0.1		
						Peptococcaceae	0.7	*Desulfurispora*	0.2	*thermophilia*	0.2
								Desulfotomaculum	0.2	*indisum*	0.1
										thermoacrtoxidans	0.02
								Sporotomaculum	0.1	*syntrophicum*	0.1
								Desulfosporosinus	0.1	*lacus*	0.04
										Unclassified	0.1
								Pelotomaculum	0.03	*isophthalicicum*	0.03
								Other	0.03		
						Veillonellaceae	0.4	*Megaspharea*	0.2	*hominis*	0.2
								Dialister	0.04	*invisus*	0.04
								Selenomonas	0.01	*infelix*	0.01
								Other	0.02		
								Unclassified	0.04		
						Ruminococcaceae	0.3	*Ruminococcus*	0.1	Other	0.01
										Unclassified	0.1
								Faecalibacterium	0.1	*prausnitzii*	0.04
										Unclassified	0.1
								Oscillospira	0.1	*eae*	0.02
										guilliermondi	0.01
										Unclassified	0.1
						Other	0.3				
						Unclassified	0.4				
				Thermoanaerobacterales	2.8	Thermoanaerobacteraceae	2.7	*Tepidanaerobacter*	1.0	*syntrophicus*	1.0
								Moorella	0.4	*glycerini*	0.3
										Unclassified	0.1
								Other	0.2		
						Other	0.1				
				Other	0.4						
				Unclassified	0.8						

		Bacilli	4.1	Bacillales	3.4	Paenibacillaceae	2.1	*Cohnella*	1.9	*soli*	1.9
										Other	0.04
								Paenibacillus	0.2	Other	0.01
										Unclassified	0.1
								Other	0.03		
						Bacillaceae	0.8	*Virgibacillus*	0.3	*salexigens*	0.1
										byunsanensis	0.05
										Unclassified	0.1
								Bacillus	0.3	*niacini*	0.03
										siralis	0.03
										Other	0.1
										Unclassified	0.1
								Geobacillus	0.1	Unclassified	0.1
								Lentibacillus	0.04	*salinarum*	0.04
								Other	0.01		
						Other	0.3				
						Unclassified	0.3				
				Lactobacillales	0.4	Leuconostocaceae	0.2	*Oenococcus*	0.2	Unclassified	0.2
								Weissella	0.03	*thailandesis*	0.02
										Unclassified	0.01
								Other	0.01		
						Streptococcaceae	0.1	*Streptococcus*	0.1	*vestibularis*	0.03
										thermophilus	0.02
										peroris	0.01
										Other	0.02
										Unclassified	0.04
						Other	0.04				
				Other	0.3						
		Unclassified	1.2								
Other	15.3										
